# Supplementary material for: Pangenome-wide and molecular evolution analyses of the Pseudomonas aeruginosa species
Source: BMC Genomics. 2016 Jan 12;17:45. doi: 10.1186/s12864-016-2364-4 (PMC4710005; doi:10.1186/s12864-016-2364-4)
Supplement: Additional file 1: — Metadata associated with the 181 strains used in this study to reconstruct the P. aeruginosa pangenome. All the information regarding the genome size, ORF predicted, RNA genes, host, geographic location, virulence, and resistance genes were recovered from PATRIC database. NA indicates no information available. (PDF 808 kb) [file 12864_2016_2364_MOESM1_ESM.pdf]

Additional File 1. Metadata associated with the 181 strains used in this study to reconstruct the P. aeruginosa pangenome. All the information regarding the genome size, ORF predicted, RNA genes, host, geographic location, virulence, and resistance genes were recovered from PATRIC database. NA indicates no information available.

Strain	Assembly length (bp)	CDS-PATRIC (Genes)	rRNAs PATRIC	tRNAs PATRIC	Isolation Country	Host	ARDB Genes	VFDB Genes	Human Homologues	Antimicrobial Resistance
**Pseudomonas_aeruginosa_138244**	6,295,127	6,015	10	55	Portugal	Homo sapiens	18	180	73	NA
**Pseudomonas_aeruginosa_152504**	6,778,878	6,298	9	63	Portugal	Homo sapiens	17	180	82	NA
**Pseudomonas_aeruginosa_18A**	6,076,446	5,690	15	59		Homo sapiens	16	182	82	NA
**Pseudomonas_aeruginosa_19660**	6,733,087	6,286	12	65	USA	Homo sapiens	17	188	80	Susceptible
**Pseudomonas_aeruginosa_19BR**	6,742,964	6,316	12	64			19	189	85	NA
**Pseudomonas_aeruginosa_213BR**	6,719,211	6,282	12	64			20	188	84	NA
**Pseudomonas_aeruginosa_2192**	6,905,121	6,490	1	44		Homo sapiens	19	195	92	NA
**Pseudomonas_aeruginosa_39016**	6,866,064	6,266	3	58		Homo sapiens	18	174	77	NA
**Pseudomonas_aeruginosa_6077**	6,967,437	6,503	20	67	USA	Homo sapiens	24	185	81	Resistant / Susceptible
**Pseudomonas_aeruginosa_62**	6,497,693	6,086	17	65	USA		17	188	80	Susceptible
**Pseudomonas_aeruginosa_9BR**	6,849,861	6,424	12	64			21	189	84	NA
**Pseudomonas_aeruginosa_AES-1R**	5,957,086	5,705	3	44		Homo sapiens	17	170	78	NA
**Pseudomonas_aeruginosa_AH16**	6,746,091	6,364	7	59	China	Homo sapiens	14	181	77	NA
**Pseudomonas_aeruginosa_ATCC_14886**	6,345,206	5,942	3	50			17	183	80	NA
**Pseudomonas_aeruginosa_ATCC_15442**	6,729,708	6,349	12	63			17	190	85	NA
**Pseudomonas_aeruginosa_ATCC_25324**	6,886,445	6,597	2	53			17	187	76	NA
**Pseudomonas_aeruginosa_ATCC_700888**	6,700,836	6,318	3	53			17	182	75	NA
**Pseudomonas_aeruginosa_B136-33**	6,421,010	5,952	12	63			17	185	80	NA
**Pseudomonas_aeruginosa_B3-1811**	6,593,639	6,203	5	53			17	185	78	NA
**Pseudomonas_aeruginosa_B3-208**	6,627,380	6,248	4	47			17	186	78	NA
**Pseudomonas_aeruginosa_B3-20M**	6,533,291	6,165	2	49			17	173	76	NA
**Pseudomonas_aeruginosa_B3-CFI**	6,677,338	6,297	5	49			17	185	76	NA
**Pseudomonas_aeruginosa_BL01**	6,389,649	5,936	12	61	USA	Homo sapiens	17	176	80	Susceptible
**Pseudomonas_aeruginosa_BL02**	6,897,031	6,467	16	66	USA	Homo sapiens	17	188	79	Susceptible
**Pseudomonas_aeruginosa_BL03**	7,004,011	6,552	12	65	USA	Homo sapiens	17	182	87	Resistant / Susceptible
**Pseudomonas_aeruginosa_BL04**	7,319,902	6,872	16	68	USA	Homo sapiens	17	190	90	Susceptible
**Pseudomonas_aeruginosa_BL05**	6,354,419	5,887	11	59	USA	Homo sapiens	17	184	81	Susceptible/Intermediate
**Pseudomonas_aeruginosa_BL06**	6,979,166	6,543	12	58	USA	Homo sapiens	17	193	90	Susceptible
**Pseudomonas_aeruginosa_BL07**	6,486,089	6,061	12	63	USA	Homo sapiens	17	188	79	Resistant/Susceptible/Intermediate
**Pseudomonas_aeruginosa_BL08**	6,944,998	6,479	12	64	USA	Homo sapiens	18	184	82	Susceptible
**Pseudomonas_aeruginosa_BL09**	6,864,557	6,391	15	68	USA	Homo sapiens	17	188	79	Susceptible
**Pseudomonas_aeruginosa_BL10**	6,489,224	6,023	11	61	USA	Homo sapiens	17	180	81	Susceptible
**Pseudomonas_aeruginosa_BL11**	6,994,234	6,563	12	64	USA	Homo sapiens	17	193	90	Susceptible
**Pseudomonas_aeruginosa_BL12**	7,406,748	6,998	12	65	USA	Homo sapiens	17	193	80	Resistant / Susceptible
**Pseudomonas_aeruginosa_BL13**	7,168,867	6,715	11	63	USA	Homo sapiens	17	187	80	Susceptible
**Pseudomonas_aeruginosa_BL14**	7,099,468	6,532	15	65	USA	Homo sapiens	17	186	82	Susceptible/Intermediate
**Pseudomonas_aeruginosa_BL15**	6,300,060	5,825	14	65	USA	Homo sapiens	17	178	79	Susceptible
**Pseudomonas_aeruginosa_BL16**	6,777,576	6,337	15	66	USA	Homo sapiens	17	184	80	Susceptible
**Pseudomonas_aeruginosa_BL17**	6,865,653	6,385	12	63	USA	Homo sapiens	19	184	81	Resistant/Susceptible/Intermediate
**Pseudomonas_aeruginosa_BL18**	6,482,158	6,059	12	60	USA	Homo sapiens	17	186	79	Susceptible/Intermediate
**Pseudomonas_aeruginosa_BL19**	6,359,545	5,917	12	64	USA	Homo sapiens	17	185	80	Susceptible
**Pseudomonas_aeruginosa_BL20**	6,696,209	6,201	15	65	USA	Homo sapiens	16	187	81	Susceptible
**Pseudomonas_aeruginosa_BL21**	6,806,078	6,344	12	64	USA	Homo sapiens	17	184	85	Susceptible
**Pseudomonas_aeruginosa_BL22**	6,936,403	6,490	17	65	USA	Homo sapiens	18	187	81	Susceptible
**Pseudomonas_aeruginosa_BL23**	7,080,433	6,621	13	65	USA	Homo sapiens	17	188	83	Susceptible
**Pseudomonas_aeruginosa_BL24**	6,900,257	6,425	11	63	USA	Homo sapiens	17	181	80	Susceptible
**Pseudomonas_aeruginosa_BL25**	6,526,145	6,075	15	65	USA	Homo sapiens	17	185	80	Susceptible
**Pseudomonas_aeruginosa_BWHPSA001**	6,406,943	5,990	14	61	USA	Homo sapiens	17	188	79	Resistant/Susceptible/Intermediate
**Pseudomonas_aeruginosa_BWHPSA002**	6,915,491	6,426	13	67	USA	Homo sapiens	17	180	81	Resistant / Susceptible
**Pseudomonas_aeruginosa_BWHPSA003**	6,819,419	6,339	15	64	USA	Homo sapiens	17	181	81	Resistant/Susceptible/Intermediate

**Pseudomonas_aeruginosa_BWHPSA004**	6,273,839	5,811	12	63	USA	Homo sapiens	17	180	80	Susceptible/Intermediate
**Pseudomonas_aeruginosa_BWHPSA006**	6,930,020	6,478	12	65	USA	Homo sapiens	17	186	80	Resistant/Susceptible/Intermediate
**Pseudomonas_aeruginosa_BWHPSA007**	6,749,180	6,336	13	62	USA	Homo sapiens	18	189	91	Resistant/Susceptible/Intermediate
**Pseudomonas_aeruginosa_BWHPSA008**	6,466,249	6,076	12	61	USA	Homo sapiens	17	182	79	Resistant/Susceptible/Intermediate
**Pseudomonas_aeruginosa_BWHPSA009**	6,409,965	5,960	13	63	USA	Homo sapiens	17	186	79	Susceptible
**Pseudomonas_aeruginosa_BWHPSA010**	6,854,990	6,483	18	70	USA	Homo sapiens	17	183	79	Susceptible
**Pseudomonas_aeruginosa_BWHPSA011**	7,042,470	6,613	12	63	USA	Homo sapiens	18	185	79	Susceptible
**Pseudomonas_aeruginosa_BWHPSA012**	6,475,264	6,020	12	63	USA	Homo sapiens	17	179	80	Resistant / Susceptible
**Pseudomonas_aeruginosa_BWHPSA013**	6,678,338	6,216	12	63	USA	Homo sapiens	17	180	85	Susceptible
**Pseudomonas_aeruginosa_BWHPSA014**	6,279,266	5,824	12	65	USA	Homo sapiens	17	181	80	Susceptible
**Pseudomonas_aeruginosa_BWHPSA015**	6,413,511	5,956	12	61	USA	Homo sapiens	17	184	80	Resistant/Susceptible/Intermediate
**Pseudomonas_aeruginosa_BWHPSA016**	6,438,821	6,056	14	64	USA	Homo sapiens	17	192	79	Susceptible
**Pseudomonas_aeruginosa_BWHPSA017**	6,904,019	6,409	12	65	USA	Homo sapiens	17	181	81	Resistant / Susceptible
**Pseudomonas_aeruginosa_BWHPSA018**	6,599,115	6,165	12	63	USA	Homo sapiens	17	182	79	Resistant/Susceptible/Intermediate
**Pseudomonas_aeruginosa_BWHPSA019**	6,407,594	5,964	12	61	USA	Homo sapiens	17	179	80	Resistant / Susceptible
**Pseudomonas_aeruginosa_BWHPSA020**	6,522,963	6,048	15	60	USA	Homo sapiens	17	185	80	Susceptible
**Pseudomonas_aeruginosa_BWHPSA021**	6,478,964	6,019	14	63	USA	Homo sapiens	17	180	80	Resistant / Susceptible
**Pseudomonas_aeruginosa_BWHPSA022**	6,858,313	6,502	15	68	USA	Homo sapiens	17	186	79	Susceptible
**Pseudomonas_aeruginosa_BWHPSA023**	6,893,268	6,449	12	60	USA	Homo sapiens	17	185	79	Susceptible
**Pseudomonas_aeruginosa_BWHPSA024**	6,743,186	6,335	12	61	USA	Homo sapiens	17	183	80	Susceptible
**Pseudomonas_aeruginosa_BWHPSA025**	6,322,238	5,860	15	65	USA	Homo sapiens	17	182	81	Susceptible/Intermediate
**Pseudomonas_aeruginosa_BWHPSA026**	6,792,144	6,327	15	65	USA	Homo sapiens	18	184	80	Resistant/Susceptible/Intermediate
**Pseudomonas_aeruginosa_BWHPSA027**	6,865,043	6,405	14	66	USA	Homo sapiens	17	185	80	Susceptible
**Pseudomonas_aeruginosa_BWHPSA028**	7,423,732	6,881	12	68	USA	Homo sapiens	17	189	81	Susceptible
**Pseudomonas_aeruginosa_C20**	6,877,672	6,459	12	58	USA	Homo sapiens	17	185	79	Susceptible
**Pseudomonas_aeruginosa_C23**	6,877,237	6,453	12	60	USA	Homo sapiens	17	185	79	Susceptible
**Pseudomonas_aeruginosa_C3719**	6,222,097	5,950	3	40	USA	Homo sapiens	20	184	86	NA
**Pseudomonas_aeruginosa_C40**	6,600,279	6,183	13	61	USA	Homo sapiens	17	187	80	Susceptible
**Pseudomonas_aeruginosa_C41**	6,774,357	6,351	12	64	USA	Homo sapiens	17	188	79	Susceptible
**Pseudomonas_aeruginosa_C48**	6,384,855	5,942	11	63	USA	Homo sapiens	17	184	80	Susceptible
**Pseudomonas_aeruginosa_C51**	6,901,793	6,409	12	63	USA	Homo sapiens	17	185	80	Susceptible
**Pseudomonas_aeruginosa_C52**	7,070,139	6,569	13	64	USA	Homo sapiens	17	186	81	Susceptible
**Pseudomonas_aeruginosa_c7447m**	6,262,305	5,805	13	63		Homo sapiens	17	192	81	NA
**Pseudomonas_aeruginosa_CF127**	6,997,351	6,471	14	66	USA	Homo sapiens	17	184	81	Susceptible
**Pseudomonas_aeruginosa_CF18**	6,486,304	6,053	14	63	USA	Homo sapiens	17	185	81	Susceptible
**Pseudomonas_aeruginosa_CF27**	6,495,953	6,008	9	61	USA	Homo sapiens	17	187	81	Resistant / Susceptible
**Pseudomonas_aeruginosa_CF5**	6,330,289	5,931	12	61	USA	Homo sapiens	18	186	81	Resistant/Susceptible/Intermediate
**Pseudomonas_aeruginosa_CF614**	6,760,825	6,314	16	67	USA	Homo sapiens	17	190	87	NA
**Pseudomonas_aeruginosa_CF77**	7,009,645	6,481	12	65	USA	Homo sapiens	17	184	80	NA
**Pseudomonas_aeruginosa_CI27**	6,743,776	6,329	4	59		Homo sapiens	17	179	78	NA
**Pseudomonas_aeruginosa_CIG1**	6,439,387	6,130	2	51		Homo sapiens	18	186	77	NA
**Pseudomonas_aeruginosa_DHS01**	6,946,419	6,604	5	62	France	Homo sapiens	20	181	79	NA
**Pseudomonas_aeruginosa_DHS29**	7,080,737	6,702	4	56	France	Homo sapiens	19	190	79	NA
**Pseudomonas_aeruginosa_DK2**	6,402,658	5,984	12	63		Homo sapiens	18	183	79	NA
**Pseudomonas_aeruginosa_DQ8**	6,703,078	6,425	2	54	China		18	193	79	NA
**Pseudomonas_aeruginosa_E2**	6,319,612	5,870	3	51		Tomato	17	186	80	NA
**Pseudomonas_aeruginosa_JD303**	6,032,077	5,782	9	28			15	182	80	NA
**Pseudomonas_aeruginosa_JD304**	6,116,697	5,919	7	31			17	186	80	NA
**Pseudomonas_aeruginosa_JD306**	6,167,835	6,048	11	26			17	179	77	NA
**Pseudomonas_aeruginosa_JD310**	6,096,124	5,923	8	32			18	181	82	NA
**Pseudomonas_aeruginosa_JD312**	5,992,607	5,804	11	31			17	185	80	NA
**Pseudomonas_aeruginosa_JD313**	5,957,709	5,715	8	30			15	178	83	NA
**Pseudomonas_aeruginosa_JD314**	5,864,640	5,657	9	29			15	181	78	NA
**Pseudomonas_aeruginosa_JD315**	6,108,864	5,917	7	29			18	185	85	NA
**Pseudomonas_aeruginosa_JD316**	5,933,692	5,681	8	32			17	182	79	NA
**Pseudomonas_aeruginosa_JD317**	6,220,844	5,996	9	31			19	187	82	NA
**Pseudomonas_aeruginosa_JD318**	5,905,973	5,680	8	28			15	183	81	NA
**Pseudomonas_aeruginosa_JD320**	6,108,204	5,829	5	29			16	177	83	NA

**Pseudomonas_aeruginosa_JD322**	6,196,416	5,983	6	31			18	188	83	NA
**Pseudomonas_aeruginosa_JD323**	5,689,853	5,504	8	31			16	184	79	NA
**Pseudomonas_aeruginosa_JD324**	6,070,901	5,939	6	31			14	185	76	NA
**Pseudomonas_aeruginosa_JD325**	5,943,714	5,707	8	31			17	181	78	NA
**Pseudomonas_aeruginosa_JD326**	6,146,463	5,951	9	30			18	184	84	NA
**Pseudomonas_aeruginosa_JD328**	6,067,306	5,802	9	31			17	180	86	NA
**Pseudomonas_aeruginosa_JD329**	6,113,201	5,936	8	29			19	189	87	NA
**Pseudomonas_aeruginosa_JD331**	5,948,930	5,691	4	30			17	176	85	NA
**Pseudomonas_aeruginosa_JD332**	5,853,818	5,642	9	30			14	180	81	NA
**Pseudomonas_aeruginosa_JD333**	6,052,680	5,802	7	30			18	181	82	NA
**Pseudomonas_aeruginosa_JD334**	6,374,685	6,137	7	27			15	183	87	NA
**Pseudomonas_aeruginosa_JD335**	6,108,256	5,897	5	30			17	185	81	NA
**Pseudomonas_aeruginosa_JJ692**	6,734,255	6,256	13	62			18	184	80	NA
**Pseudomonas_aeruginosa_LCT-PA102**	6,695,513	6,283	2	53			14	184	78	NA
**Pseudomonas_aeruginosa_LCT-PA220**	6,700,845	6,305	3	53	China	Homo sapiens	14	183	78	NA
**Pseudomonas_aeruginosa_LCT-PA41**	6,699,635	6,311	2	51	China	Homo sapiens	14	183	78	NA
**Pseudomonas_aeruginosa_LES431**	6,550,070	6,135	12	66			18	190	80	NA
**Pseudomonas_aeruginosa_LESB58**	6,601,757	6,137	8	68	United Kingdom		18	189	80	NA
**Pseudomonas_aeruginosa_M18**	6,327,754	5,892	12	64			17	184	79	NA
**Pseudomonas_aeruginosa_M8A1**	6,347,003	5,885	12	63			17	187	80	Susceptible
**Pseudomonas_aeruginosa_M8A2**	6,344,408	5,906	15	66			17	185	81	Susceptible
**Pseudomonas_aeruginosa_M8A3**	6,330,876	5,882	12	64			17	184	81	Susceptible
**Pseudomonas_aeruginosa_M8A4**	6,346,902	5,929	9	60			17	188	80	Susceptible
**Pseudomonas_aeruginosa_M9A1**	6,391,987	5,943	12	64			17	183	80	Susceptible
**Pseudomonas_aeruginosa_MPAO1P1**	6,231,994	5,845	7	62			17	190	78	NA
**Pseudomonas_aeruginosa_MPAO1P2**	6,207,826	5,831	10	57			17	190	79	NA
**Pseudomonas_aeruginosa_MRW441**	6,250,825	5,867	9	57			18	191	77	NA
**Pseudomonas_aeruginosa_MSH-10**	6,464,013	6,041	14	66			17	188	79	NA
**Pseudomonas_aeruginosa_MSH10**	6,462,049	6,049	14	62	USA		17	187	79	Susceptible
**Pseudomonas_aeruginosa_MSH3**	6,467,380	6,059	13	64	USA		17	188	79	Susceptible
**Pseudomonas_aeruginosa_N002**	5,634,227	6,581	3	28	India		22	197	104	NA
**Pseudomonas_aeruginosa_NCGM2S1**	6,764,661	6,325	12	66	Japan	Homo sapiens	19	185	80	NA
**Pseudomonas_aeruginosa_NCMG1179**	6,688,270	6,254	4	58	Japan	Homo sapiens	19	196	78	NA
**Pseudomonas_aeruginosa_PA14**	6,508,740	6,039	12	62			17	184	80	NA
**Pseudomonas_aeruginosa_PA1**	6,528,877	6,117	12	65			17	184	79	NA
**Pseudomonas_aeruginosa_PA1R**	6,309,305	5,916	12	65			14	183	71	NA
**Pseudomonas_aeruginosa_PA21_ST175**	6,853,852	6,490	4	58	Spain	Homo sapiens	18	191	77	NA
**Pseudomonas_aeruginosa_PA45**	6,592,856	6,171	3	57	Italy	Homo sapiens	17	183	80	NA
**Pseudomonas_aeruginosa_PA7**	6,588,339	6,064	8	65		Homo sapiens	17	141	82	NA
**Pseudomonas_aeruginosa_PAb1**	5,984,633	5,745	10	63		Homo sapiens	16	177	81	NA
**Pseudomonas_aeruginosa_PABL056**	7,225,986	6,813	7	61	USA	Homo sapiens	20	183	80	NA
**Pseudomonas_aeruginosa_PACS2**	6,492,423	6,016	8	65			17	184	81	NA
**Pseudomonas_aeruginosa_PADK2_CF510**	6,683,163	6,315	12	63	Demark	Homo sapiens	18	185	82	NA
**Pseudomonas_aeruginosa_PAK**	6,381,849	5,929	15	65			17	187	79	NA
**Pseudomonas_aeruginosa_PAO1-CipR**	5,512,595	5,382	8	55			14	165	86	NA
**Pseudomonas_aeruginosa_PAO1**	6,264,404	5,836	8	64			17	192	79	NA
**Pseudomonas_aeruginosa_PAO1-VE13**	6,265,484	5,833	13	63			17	191	79	NA
**Pseudomonas_aeruginosa_PAO1-VE2**	6,265,484	5,837	13	63			17	191	79	NA
**Pseudomonas_aeruginosa_PAO579**	6,012,534	5,658	14	63	Australia	Homo sapiens	15	185	78	NA
**Pseudomonas_aeruginosa_PAO581**	6,043,974	5,655	13	63			16	185	78	NA
**Pseudomonas_aeruginosa_PFK10**	6,033,935	6,083	10	54	India		21	190	94	NA
**Pseudomonas_aeruginosa_PGPR2**	6,687,777	6,648	12	68	India		22	188	89	NA
**Pseudomonas_aeruginosa_PK6**	6,038,536	5,955	4	55	India		19	175	84	NA
**Pseudomonas_aeruginosa_RP73**	6,342,034	5,940	12	63		Homo sapiens	18	150	79	NA
**Pseudomonas_aeruginosa_S35004**	6,962,206	6,446	12	64	USA	Homo sapiens	17	184	81	Resistant/Susceptible/Intermediate
**Pseudomonas_aeruginosa_S54485**	7,002,166	6,618	12	63	USA	Homo sapiens	17	184	78	Resistant / Susceptible
**Pseudomonas_aeruginosa_SCV20265**	6,725,183	6,312	12			Homo sapiens	17	182	79	NA

**Pseudomonas_aeruginosa_SJTD-1**	6,063,872	5,676	12	63	China		17	180	79	NA
**Pseudomonas_aeruginosa_str_Stone_130**	7,420,589	6,999	9	65			20	191	81	NA
**Pseudomonas_aeruginosa_U2504**	7,046,941	6,631	12	64	USA	Homo sapiens	21	184	80	Resistant / Susceptible
**Pseudomonas_aeruginosa_UCBPP-PA14**	6,537,648	6,022	8	63		Homo sapiens	17	185	80	NA
**Pseudomonas_aeruginosa_UDL**	6,286,208	5,843	12	64	USA	Homo sapiens	17	190	80	Susceptible/Intermediate
**Pseudomonas_aeruginosa_VRFPA01**	5,929,762	6,682	3	54	India	Homo sapiens	28	138	111	NA
**Pseudomonas_aeruginosa_VRFPA02**	6,474,922	6,496	5	58	India	Homo sapiens	19	189	90	NA
**Pseudomonas_aeruginosa_VRFPA03**	7,037,729	7,170	9	57	India	Homo sapiens	28	188	90	NA
**Pseudomonas_aeruginosa_VRFPA04**	6,998,695	6,654	16	58	India	Homo sapiens	21	181	83	NA
**Pseudomonas_aeruginosa_VRFPA05**	7,050,363	6,802	18	66	India	Homo sapiens	21	192	89	NA
**Pseudomonas_aeruginosa_VRFPA07**	7,177,216	6,848	11	57	India	Homo sapiens	17	190	87	NA
**Pseudomonas_aeruginosa_VRFPA08**	7,023,037	6,666	14	65	India	Homo sapiens	20	184	82	NA
**Pseudomonas_aeruginosa_WC55**	6,775,564	6,425	2	30	USA		17	185	79	NA
**Pseudomonas_aeruginosa_X13273**	7,037,692	6,634	11	63	USA	Homo sapiens	17	184	76	Resistant / Susceptible
**Pseudomonas_aeruginosa_X24509**	6,415,963	5,954	14	64	USA	Homo sapiens	17	189	80	Resistant / Susceptible
**Pseudomonas_aeruginosa_XMG**	6,413,604	5,960	3	58	China		17	183	79	NA
